# Current Evidence for Stereotactic Body Radiotherapy in Lung Metastases

**DOI:** 10.3390/curroncol28040233

**Published:** 2021-07-15

**Authors:** Enrique Gutiérrez, Irving Sánchez, Omar Díaz, Adrián Valles, Ricardo Balderrama, Jesús Fuentes, Brenda Lara, Cipatli Olimón, Víctor Ruiz, José Rodríguez, Luis H. Bayardo, Matthew Chan, Conrad J. Villafuerte, Jerusha Padayachee, Alexander Sun

**Affiliations:** 1 Princess Margaret Cancer Centre, Radiation Medicine Program, University Health Network, Toronto, ON M5G2M9, Canada; Enrique.Gutierrez@rmp.uhn.ca (E.G.); matthew.chan@bccancer.bc.ca (M.C.); Conrad.Villafuerte@rmp.uhn.ca (C.J.V.); Jerusha.Padayachee@rmp.uhn.ca (J.P.); 2 Department of Radiation Oncology, University of Toronto, Toronto, ON M5G2M9, Canada; 3 Western National Medical Center, Department of Radiation Oncology, Mexican Institute of Social Security (IMSS), Belisario Domínguez 1000, Guadalajara 44340, Jalisco, Mexico; irvingsanchez.md@gmail.com (I.S.); omardiazcazares@gmail.com (O.D.); advaqui@hotmail.com (A.V.); ricardoivan.balderrama@gmail.com (R.B.); drfuentes@protonmail.com (J.F.); brendalara912@hotmail.com (B.L.); drolimon@hotmail.com (C.O.); victormjrp5@gmail.com (V.R.); Jose_2906@hotmail.com (J.R.); luis.bayardo@imss.gob.mx (L.H.B.)

**Keywords:** lung SBRT, oligometastatic disease, lung cancer, lung metastases

## Abstract

Lung metastases are the second most common malignant neoplasms of the lung. It is estimated that 20–54% of cancer patients have lung metastases at some point during their disease course, and at least 50% of cancer-related deaths occur at this stage. Lung metastases are widely accepted to be oligometastatic when five lesions or less occur separately in up to three organs. Stereotactic body radiation therapy (SBRT) is a noninvasive, safe, and effective treatment for metastatic lung disease in carefully selected patients. There is no current consensus on the ideal dose and fractionation for SBRT in lung metastases, and it is the subject of study in ongoing clinical trials, which examines different locations in the lung (central and peripheral). This review discusses current indications, fractionations, challenges, and technical requirements for lung SBRT.

## 1. Introduction

Historically the standard treatment for metastatic disease has been with systemic therapy alone. However, more recent evidence from the retrospective and prospective series indicates improved long-term results when patients are managed with the addition of aggressive local treatments [1,2,3,4,5,6,7,8,9]

Lung metastases develop in 20–54% of cancer patients at some point in their disease [10]. At least 50% of cancer-related deaths occur at this stage. Budczies et al. conducted a cohort study in 1008 postmortem patients, and the lung was the third most frequent site of metastases of 16 types of solid cancer [11]. Some authors consider it to be the second most frequent site [10,11] of metastatic target for neoplastic cells since it has a microenvironment that is rich in vascular supply, as well as containing small capillaries with a very short distance to the intravascular space [12]. In 1889, Paget proposed that metastatic disease does not progress randomly but in a process that has recently been termed the metastatic cascade [12]. This process includes the steps of angiogenesis, intravasation, survival in circulation, extravasation, establishment, and growth [13,14].

Cancer cells migrate towards higher-oxygen areas, which can be achieved through the epithelial-mesenchymal transition, orientation, interaction within the stroma, and evasion of the immune system [13].

As the tumor grows, physical barriers, such as the basement membrane and interstitial connective tissue, must degrade for tumor invasion to occur. Proteolytic enzymes (e.g., collagenase, trypsin, plasmin, and cathepsin B) and matrix metalloprotease expressed in tumor cells facilitate tumor spread by denaturalizing the extracellular matrix, migration, and chemotaxis, also called intravasation [10]. Hematogenic spread occurs mainly through the venous drainage system and the pulmonary arteries (but is less common through the bronchial arteries) [13]. Lymphatic spread and tracheobronchial spread are unusual, accounting for 2–5% of pulmonary metastases, and direct extension is the least common pathway of all [10].

Finally, the proliferation of metastatic foci is succeeded by the production of proangiogenic factors such as vascular endothelial growth factor, fibroblast growth factor, and interleukin-8 (IL-8) [14].

## 2. Definition of Oligometastatic Disease

The term oligometastasis first appeared in 1995 and defines a state between localized and widely disseminated disease [15,16]. This concept was consolidated by integrating the elements of the size and number of lesions that occur synchronously (at diagnosis of the primary tumor or up to 3 months later) or after treatment [17,18]. An oligometastatic status indicates the candidacy for radical treatment for both primary and metastatic tumors [7,19,20].

Another scenario in which the benefit of treatment is being analyzed is oligoprogression, a state where only a limited number of metastases progress, while the rest remain stable or respond to systemic treatment [19,21]. The treatment options are to change systemic therapy, continue with a given same scheme (in minimal progression) or delay changing the systemic therapy by adding local treatment, such as stereotactic body radiation therapy (SBRT) [19,21].

## 3. Number of Metastases

Oligometastatic disease (OMD) has been defined as having up to five lesions occurring separately and distributed in up to three organs [22]. There is no standardized definition, but the SABR-COMET protocol adopts this definition for its inclusion criteria, indicating also that the maximum number of metastases per organ must be equal to or less than three lesions [23]. Multiple retrospective studies have also used a cut-off point of five lesions [7,24,25,26,27], including patients with extrathoracic metastasis, who were candidates for the treatment with radical intent [24,25]. However, other studies, including ongoing studies have included up to 10 lesions [28]. Schanne et al. analyzed patients with Non-small cell lung cancer (NSCLC) and oligometastatic disease across 54 studies where 90.5% of patients had a single metastatic lesion. Most studies have described in different ways the term OMD as it has been defined with significant variation regarding the size and the number of lesions [22]. Therefore, the term OMD continues to be defined. Although the number of metastatic lesions included has varied among different protocols, most trials have identified three or fewer metastases. Consequently, this number could serve as a cut-off point that guides radical management in OMD in the future [5,23,27,29].

Yamamoto et al. concluded that patients with more than five lesions could be treated, as long as dose constraints are achieved. However, in that study, most patients (74%) had a unique lesion [27]. Another critical factor is lesion size. Rusthoven et al. used a cut-off point of 7 cm per lesion [30] and Salame et al. included patients with lesions up to 10 cm or 500 cm^3^ [26].

## 4. Diagnosis and Imaging of Lung Metastases

### Imaging Studies

A chest X-ray is a low-sensitivity tool that cannot detect lesions of less than 1 cm [31]. In patients with extrathoracic cancer, detection of a new nodule on an X-ray has a 25% probability of indicating a metastatic lesion [32].

**Figure 1:**
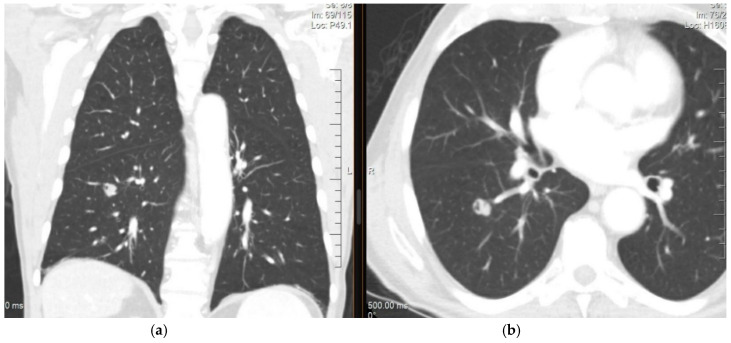
CT of the chest (**a**) coronal view and (**b**) axial view showing a nodule in the right lower lobe measuring 1.5 cm, lobulated and cavitated. The lesion was biopsied and revealed an adenocarcinoma with cytomorphology and an immunoprofile that was consistent with a colorectal primary.

Chest computed tomography (CT) with slices of 1–1.25 mm is the preferred approach for assessing lungs to evaluate and determine the size of the lesion [27]. The characteristics of a metastatic lesion are widely variable. A new nodule > 10 mm has a 15% chance of malignancy, as shown in Figure 1. However, regardless of its size, an extrathoracic primary with a new lung lesion is highly suggestive of metastases. Malignant lesions commonly occur with spiculated and irregular margins. However, metastases should not be ruled out if the lesions are rounded and smooth [33].

Fluorodeoxyglucose-positron emission tomography (FDG-PET) is a highly sensitive approach for the detection of malignant lesions with solid characteristics and larger than 8 mm, showing a sensitivity of 89% (95% CI, 86–91%) and a specificity of 75% (95% CI, 71–79%) [34]. The capacity of FDG-PET to detect pulmonary metastases and its relation to the treatment results of lung SBRT can be characterized by SUVmax, SUVmedian, and metabolic tumor volume (MTV). In a study by Mazzola et al., prior to the SBRT treatment, the mean SUVmax was 6.5 (range 4–17), the mean SUVmedian was 3.7 (range 2.5–6.5), and the mean MTV was 2.3 (range 0.2–31) cm^3^. A SUVmax < 5 and a SUVmedian < 3.5 were associated with a full response at 6 months [35]. Among patients who underwent metastasectomy, those with SUVmax > 4.5 (51.6% vs. 74.0%) had a decreased 5-year overall survival [36]. According to Mayo Clinic and Department of Veterans Affairs (VA) models, another relevant tool is the pre-test probability of malignancy based on clinical characteristics and radiographic findings (age, smoking history, history of extrathoracic cancer, the diameter of the nodule in mm, and localization within the upper lobe) [37]. If PET-scan results are negative and the pre-test probability is equal to or greater than 65%, a needle-biopsy or video-assisted thoracic surgery should be considered [38]. In patients with an indeterminate solitary pulmonary nodule (SPN) > 8–10 mm in diameter, a biopsy is recommended in the following situations: (1) Pre-test probability and radiographic findings are discordant, (2) benign diagnosis is suspected, and (3) when a fully informed patient desires proof of a malignant diagnosis before surgery, especially when the risk for surgical complications is high [39].

The possibility of technical artifacts should be taken into account. Catheters, metallic prostheses, and other devices can show more FDG uptake and overestimate affected areas. Another possible artifact is one generated by different respiratory phases when PET is combined with CT, being the most common site of discrepancy the lung–diaphragm limits, potentially confusing the avid areas of FDG of the liver with lung lesions [40].

Another alternative under evaluation is the use of liquid biopsies from blood samples, where nucleic acids from tumor cells can be identified [18]. Lebofsky et al. analyzed patients with recurrent/metastatic cancer and failure of standard therapy, a biopsy was compared to circulating tumor DNA determination, and a 97% match was achieved [41].

## 5. Stereotactic Body Radiation Therapy for the Lung

Stereotactic body radiation therapy (SBRT) is a noninvasive cancer treatment that uses high doses of precise radiation to extracranial target sites [42]. The American Association of Physicists in Medicine (AAPM) defines SBRT as a stereotactic treatment that delivers a high dose of radiation within a short cycle, generally limited to less than or equal to five fractions with a dose of 6 to 34 Gy per fraction [43,44]. Outside the United States, SBRT has been accepted as a highly conformal technique with regimens including up to 10 fractions with a biologically effective dose (BED) ≥ 100 Gy_10_ or doses at least biologically equivalent to a radical course of treatment when given over a protracted conventionally (1.8–3 Gy/fraction) fractionated schedule [43,45,46,47].

However, groups in several countries (AAPM, ASTRO, ACR, CARO-SBRT, and the NRIG) agree on the following points: SBRT is an external beam radiotherapy method that accurately delivers a high dose of radiation in one or a few fractions, to an extracranial target, which results in an increased effective biological dose [45,47].

### Radiobiological Principles of SBRT

The loss of reproductive ability due to the creation of double-stranded breaks in DNA is the primary mechanism by which conventional irradiation kills a cell: Any cell that is unable to reproduce indefinitely is considered to be dead by definition, although it may remain metabolically active for some time [48,49].

Five critical factors determine the effects of radiotherapy on tumors:

1. Repair of sublethal cell damage;
2. Cell repopulation followed by radiation;
3. Cell redistribution in the cell cycle;
4. Re-oxygenation of surviving cells;
5. Radiosensitivity (intrinsic) [48,49].

If a given dose of radiotherapy is divided into daily fractions according to a conventional scheme, redistribution and re-oxygenation facilitate the increase in cell death by redistributing resistant survivors into more radiosensitive phases over time. However, cell repair and repopulation produce increased numbers of surviving cells due to cell recovery and repopulation between doses [48].

Following radiation, three phases of histopathologic change in the lungs have been described. The early/latent phase occurs within a month after radiation and is characterized by the loss of type I alveolar epithelial cells (AEC), alveolar transudates, interstitial edema, and type II AEC morphologic changes. The acute exudative phase (radiation pneumonitis) occurs between three weeks and up to 6 months after radiation. This phase presents fibrin-rich exudates, interstitial edema, and accumulation of alveolar macrophages. The late or fibrotic phase begins approximately 6 months after radiation and is defined by a constant loss of type I AEC, capillary loss, and progressive collagen deposition [50].

For SBRT, there were initial queries on whether the mechanism of action of the killing of tumor cells is similar to conventional radiotherapy. Several studies have shown that the radiobiology of SBRT is quite different [51,52,53,54].

The influence of the five Rs on the treatment outcome is modified with the use of SBRT. Due to the shorter treatment time and decreased number of fractions, tumor repopulation and redistribution effects are diminished [52,55]. The steep dose gradients lessen the impact of normal organ sparing [47,52]. Reoxygenation and radiosensitivity remain as factors. However, acute hypoxia and fast reoxygenation are more commonly observed with SBRT compared to chronic hypoxia and slow reoxygenation [52,56,57,58].

Tumor cell death from SBRT can be a result of the combination of direct tumor cell eradication and indirect tumor cell killing secondary to vascular or endothelial damage [51,52,54,59,60]. Vascular damage could indirectly lead to tumor death for two reasons. SBRT can abruptly cut off blood supply and can induce endothelial apoptosis, which can heighten tumor radiosensitivity [54,61,62].

Numerous reports have described that vascular tumor damage as tumor volume decreases after being irradiated with a dose higher than 10 Gy in a single event. The vascular tumor structures become disorganized and fragmented [51,53]. Endothelial tumor cells die as a result of direct radiation damage, vascular permeability, and plasma extravasation, causing erythrocyte concentration within the narrow capillaries, leading to retardation, blood stasis, and vascular collapse [53].

Immune reactions and death of cancer stem cells are also thought to contribute to SBRT radiobiology [52,53,54]. It has been suggested that SBRT also provokes an immune reaction by increasing T-cell, leading to reduction of the primary tumor cells or distant metastases in a CD_8+_ T-cell dependent fashion [51].

Cancer stem cells are described as being perivascular. They are considered to be a possible cause of radioresistance in conventional radiotherapy as they are able to re-proliferate even after irradiation. SBRT is thought to be able to eradicate these stem cells, thereby decreasing the chances of recurrence [53,54].

## 6. Eligible Patients

Pulmonary comorbidity is the most common reason for inoperability. Patients who are considered medically inoperable are defined based on poor lung function evaluated by a thoracic surgeon or respirologist and encompassing the following parameters: Predicted forced expiratory volume in 1 s (FEV1) < 40%, predicted postoperative FEV1 < 30%, baseline hypoxemia (≤70 mmHg) and/or hypercapnia (>50 mmHg), predicted reduced diffusing capacity < 40% and predicted consumption during exercise < 50% [63,64]. Poor lung function by itself is not a contraindication for SBRT. There is no lower limit of lung function prior to SBRT treatment and may even be offered for patients with extreme pulmonary comorbidities [65].

Inoperability also encompasses serious comorbidities such as severe pulmonary hypertension; diabetes mellitus with end organ damage; cerebral vascular disease; severe chronic heart disease or severe cardiovascular disease [41,66,67] SBRT is recommended as a treatment option for this population if they have an estimated life expectancy greater than 1 year [68].

Additional criteria for eligibility include refusing surgical intervention [41,69], recurrent or metastatic lung lesions [69], Eastern Cooperative Oncology Group score ≤ 3 [67,68] or controlled primary [24,69]. There is no contraindication in terms of age [68,70] (see Table 1).

### 6.1. Considerations of the Number and Size of Metastases

In May 2020, Bernard et al. reported the case of a patient with five metastatic lung lesions that were treated synchronously with SBRT, followed by two additional lesions also treated synchronously with SBRT, for a total of seven lesions in the same lung, separated by less than 5 cm. After a 14-month follow-up, the authors found no progression and no significant late side effects [71].

In relation to the size of treatable lesions, a study by the German Society for Radio Oncology (DEGRO) established a consensual limit of lung tumor size for SBRT of 4 to 5 cm, with a more fractionated regimen for larger tumors [72].

In one of the largest series of metastatic lung tumors treated with SBRT, the experience of the RSSearch^®^ Patient Registry, the median number of metastases was 1 (1 to 3), with a mean volume of 10.58 cc (0.1 to 654.5 cc). The mean overall survival for the entire group was 26 months. The overall survival at 1, 3, and 5 years was 74.1%, 33.3%, and 21.8% of patients, respectively. The mean local control (LC) for the group was 53 months. The LC rate at 1, 3, and 5 years was 80.4%, 58.9%, and 46.2%, respectively. A statistically significant difference was identified, with improvement in local control for minor tumors. Local control at 2 years was 72.9%, 64.2%, and 45.6% for tumor volume <11 cc, 11–27 cc, and >27 cc, respectively (*p* = 0.0005 by the log-rank test; *p* = 0.0011 by the Gehan-Breslow-Wilcoxon test). This translated into an improvement in OS, with a 2-year OS of 62.4%, 60.9%, and 46.1% for tumors with volumes of <11 cc, 11–27 cc, and >27 cc, respectively, and the mean OS for lesions <11 cc, 11–27 cc, and >27 cc was 29, 31, and 21 months, respectively (*p* = 0.0023 by the log-rank test; *p* = 0.0011 by the Gehan-Breslow-Wilcoxon test) [70].

The ASTRO 2017 evidence-based guideline for NSCLC established that SBRT is an appropriate option for tumors > 5 cm in diameter within an acceptable therapeutic range. However, this applies to primary lung tumors, with conditional strength of recommendation and a low quality of evidence [42].

Few results have been produced specifically for the use of SBRT in larger lung tumors [73].

### 6.2. Peripheral and Central Lesions: Technical, Fractional, and Dose Prescription Differences

**Figure 2:**
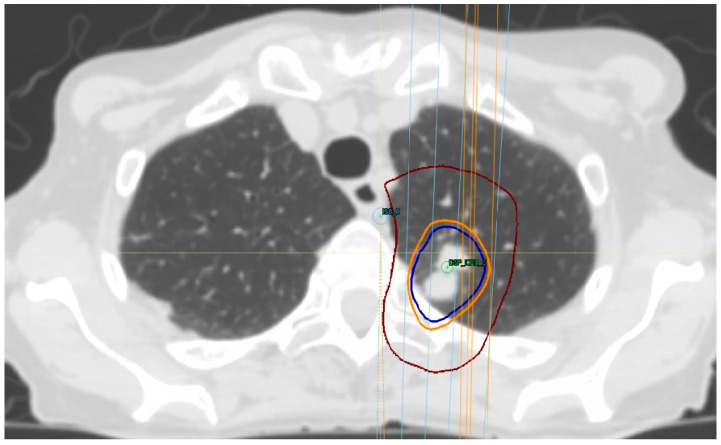
Treatment-planning dose for lung SBRT in a patient with biopsy-proven metastatic breast cancer, prescribing 48 Gy in four fractions. Isodose lines: Burgundy, 24 Gy; light orange, 44 Gy; and blue, 48 Gy.

Traditionally, only patients with tumors at least 2 cm away from the bronchial tree have been considered for SBRT, following a commonly used three to four fraction regimen [15] (Figure 2). However, at present, patients with central tumors, defined as those where the closest point is within 2 cm proximal to (but not contacting or abutting) the main bronchial tree or within 2 cm (contacting or not) of mediastinal structures [74,75], are at higher risk for toxicity when treated with SBRT, compared to patients with peripheral tumors [34], avoiding the use of a three-fraction regimen is recommended in this scenario [42]. Tumors with the highest risk for SBRT are those with an ultra-central location, defined as any GTV at ≤1 cm from the proximal bronchial tree that overlaps the trachea or the main bronchus [75] or tumors that contact (abut) the proximal bronchial tree [74,75]. High rates of toxicity and death are associated with treatment in this situation, increasing the interest in identifying an optimal, effective, and safe dose for this group of patients [76].

To reduce the probability of these complications, Bral et al. proposed a treatment with adapted risk fractions, increasing the number of fractions to the range of five to eight and with doses per fraction of 4–8 Gy, which have shown acceptable toxicity [77].

For central tumors in which SBRT is considered very high risk, hypofractionated radiotherapy using 6–15 fractions can be considered, transposing what is established by the ASTRO guideline for NSCLC in the early stages [42].

Timmerman et al. reported high rates of toxicity associated with central tumors compared to peripheral ones in the definitive treatment setting for patients with NSCLC [76]. Likewise, Lischalk et al. demonstrated a rate of local control of 57.4% at 2 years, significantly lower than that reported by Timmerman (95%) [78].

More recent retrospective data that include heterogeneous dose and fractionation schedules seem to indicate that the risk for irradiation in the central regions may not have the significant impact postulated by Timmerman et al. [76,78,79]. Chaudhuri et al. observed that this group of central tumors (including primary NSCLC and lung metastases) exhibited excellent tumor control and a similar toxicity rate to its less central counterpart, suggesting that even ultra-central tumors can be treated with 50 Gy in four or five fractions if not in contact with the esophagus [80].

The fractionation of 50 Gy in five fractions was described by Bezjak et al. in the context of early-stage NSCLC, reporting reasonable control rates and acceptable toxicities [81]. In this context, the NRG-BR001 clinical trial was designed, which is currently evaluating prescribed doses for lung metastases of 45 Gy in three fractions for peripheral lesions and 50 Gy in five fractions for central lesions. The successful completion of this trial will provide valuable information for the design of new clinical trials and the development of treatment guidelines for oligometastatic disease [82].

There is no consensus at present on the ideal dose and fractionation for SBRT in lung metastases [78,80]. A brief summary of published studies regarding differences in dose and fractionation for SBRT for lung metastases according to central or peripheral location is depicted in Table 2.

## 7. Treatment Volumes

The gross tumor volume (GTV) represents the solid tumor and ground glass density in each axial CT slice using a lung window (this value can be based on FDG-PET if available) [4,24,68]. An expansion from the GTV to the clinical target volume (CTV) margin is not routinely added for lung SBRT practice. Thus, the CTV margin is commonly 0 mm [63,64]. Grills et al. conducted a Phase II study, called Trial in Stereotactic Lung Radiotherapy, and used a 4 mm expansion (3–5 mm) of the GTV-ITV to create the CTV [65].

The creation of an internal target volume (ITV) is mandatory in this scenario [4,66,68].

The ITV can be created from an eight-phase 4D tomography acquired in a normal respiratory cycle. The GTV is outlined in a free-breathing scan, and it is expanded in four inspiratory and four expiratory phases. When the breath-hold technique is used, ITV creation is not mandatory, as it is significantly reduced compared to the free-breathing phase [4,24,66,68]. Finally, the planning target volume (PTV) is created by an isotropic growth of 5 mm from the ITV (range 3–7 mm) [4,24,66,68].

## 8. Treatment Dose

A dose and fractionation with BED of at least 100 Gy should be used [68]. According to the consensus of the ESTRO ACROP, the selection of a scheme depends upon the location and size of the tumor [68]. (see Table 3).

According to CARO, the following doses and fractionations can be used for primary and metastatic lung lesions [44]. (see Table 4).

## 9. Technical Requirements

The main technical requirements for SBRT include:

1. Modern linear accelerators to enable image-guided radiation therapy (IGRT) and motion-management systems;
2. Sophisticated immobilization devices [44,89];
3. Quality controls [72].

### 9.1. Simulation

Two critical issues in the simulation of pulmonary SBRT are the immobilization and evaluation of tumor movement [64]. Patients are normally positioned supine with their arms overhead, in a custom immobilization device [90], such as a vacuum-sealed foam bag, a stereotactic frame with a wing board and an alpha-cradle, and an immobilizer for the feet and knees [91].

The images required for simulation and planning may include detailed 4D-CT motion estimation, as well as additional soft tissue (MRI) or metabolic (PET) information [92,93]. The latter is particularly useful for tumors that are not well-defined or close to the chest wall [44].

The standard imaging modality for targeting lung tumors and organs at risk (OAR) contouring is tomography, with a maximum thickness per slice of < 3 mm, which must completely include both lungs [45,94] and at least one individual evaluation of the movement of the lungs using 4D-CT. Where this technology is lacking, the tumor movement can be determined by fluoroscopy or scanning in inspiration and expiration [45].

### 9.2. Pretreatment Setup and Treatment Delivery

#### 9.2.1. IGRT and Motion Management Systems

The available image-guided radiation therapy (IGRT) and motion management techniques are classified into three domains: Kilovoltage (kV) images, megavoltage (MV) images, and optical images [91].

Conventional kV-imagers can be fluoroscopic imaging devices, retractable kV sources, detector panels, mounted X-ray imagers, and floor detectors that provide flat radiographic images of the patient. The 3D-CT with cone-beam (3D-CBCT) shows the internal anatomy of the patient before each fraction, allowing the visualization of a range of geometric deviations, such as uncertainties due to movement [91,94]. However, for lung images, the respiratory-phase projections are averaged to reconstruct a single 3D scan, leaving blurred regions of interest or multiple diaphragmatic artifacts that give incorrect information on the tumor amplitude and its position relative to the OARs during breathing. The use of 4D-CT, by contrast, allows respiratory movement to be considered [91,95].

The 4D-CBCT provides complementary information on the interfraction trajectory of the tumor that 3D-CBCT cannot, ensuring that the margins around the target are kept small, reducing their inter-observer variability for patient positionin [15]. MV images can be obtained using electronic portal imaging devices, fan beam MV-CT with tomotherapy, MV-CBCT, and providing 3D images before treatment as a quick and accessible tool to replace dosimetry and verification of modulated deliveries [91].

Repositioning the treatment couch is another important pretreatment intervention. The patient can be positioned and aligned according to markings, tattoos or immobilization devices. After imaging, matching the patient’s current position against established landmarks is mandatory, and this can be achieved using either a couch with three translations and one rotation on the anteroposterior axis or one with 6 degrees of freedom, if available. The latter option allows for two extra rotations in the posteroanterior and lateral axes, representing clear advantages to correct positioning errors but is not mandatory for SBRT according to the available guidelines [91,96,97].

Typical strategies for managing respiratory movement include deep inspiration breath-hold during treatment, through active control of breathing, abdominal compression, and other mechanical means. Alternatively, radiation can be delivered at specific phases of the respiratory cycle using a respiratory-gated system [91,98]. Another device for the continuous administration of positive air pressure is being tested for its potential use in pulmonary radiotherapy [91,99].

These techniques are adopted to support the treatment in real time, either by moving the full linear accelerator, tilting the gantry, repositioning the patient with a robotic couch or changing the position and shape of the treatment beam with a multi-leaf collimator [95]. The most advanced motion-management technique, which is not based on images, is that of electromagnetic transponders [91,99]. Tumor tracking can also be performed using direct imaging monitoring (fluoroscopy, slow CT or 4D-CT), which is frequently associated with fiducial markers [64,100] or by the evaluation of the chest wall [101].

#### 9.2.2. Sophisticated Immobilization Devices

A wide variety of immobilization devices exist, from stereotactic body frames to alpha cradles, vacuum bags, foot, and knee supports, and abdominal compressors [44]. The choice of which to use depends upon the experience with each and availability at a given institution.

#### 9.2.3. Quality Control

To carry out adequate quality control, the following is required: Small-field dosimetry for commissioning with corresponding detectors (e.g., microchamber) [102]; system-specific end-to-end tests for both static and moving target volumes, especially if a respiratory management system is being used [103]; periodic verification of geometric and dosimetric accuracy according to system-specific guidelines; and daily quality control of the consistency of the stereotactic frame and/or the isocenter image guide system with the isocenter treatment beam. The dosimetric precision must be a maximum of 3% from a target volume of more than or equal to 2 cc with homogeneous phantoms. For target volumes smaller than 2 cc, the measurement uncertainties may be greater than the desired dosimetric precision [72].

The lung dose constraints suggested by the American Association of Physicists in Medicine (AAPM) are V7 < 1500 cc for one fraction, V11.6 < 1500 cc for three fractions (2.9 Gy/fraction), and V12.5 Gy > 1500 cc for five fractions (2.5 Gy/fraction) to an endpoint grade 3 toxicity for basic lung function and V7.4 Gy < 100 cc for one fraction, V12.4 < 1000 cc for three fractions (3.1 Gy/fraction), and V13.5 < 1000 cc for five fractions for pneumonitis [43]. The Japan Clinical Oncology Group (JCOG) 0403 Protocol considered dose constraints for the lung with a mean dose ≤ 18 Gy, 40 Gy irradiated volume ≤ 100 cc, V15Gy < 25%, and V20 < 20% [104]. Dunlap et al. [105] determined a dose constraint for the chest wall of 30 Gy in three to five fractions to < 30 cm^3^ to decrease the risk of toxicity without compromising tumor coverage.

**Figure 3:**
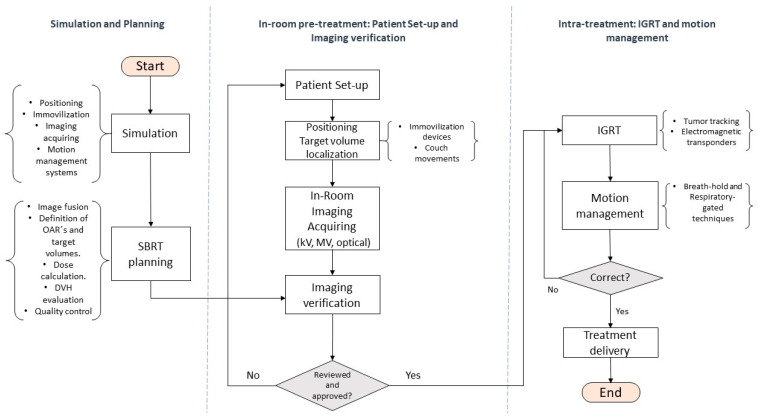
Flow chart describing the minimum technical requirements necessary to carry out the SBRT process. Local control and overall survival.

Figure 3 summarizes and describes the minimum technical requirements to carry out a SBRT protocol.

In a series of 206 patients treated with SBRT and a median follow-up of 26 months, 54% of the patients died, the median OS was 33 months, and the median PFS was 13 months. Although the median PFS survival was low at 2, 3, and 5 years (36%, 25%, and 16%, respectively), the local control was high (85%, 83%, and 81%, respectively) [106].

In another series of 219 patients with a median follow-up of 16.5 months, the median OS was 27.6 months, freedom from distant progression (DP) at 2, 3, and 5 years (46%, 40%, and 34%, respectively) were also lower than LC at 2, 3, and 5 years (84%, 78%, and 75%, respectively) [107]. In terms of histology, Takeda et al. [6] reported worse local control in patients with colorectal pulmonary metastases treated with SBRT compared to other tumors. Therefore, dose escalation should be considered in such cases. A retrospective study conducted by Jingu et al. [108] reported a better local control rate with higher BED (≥100 Gy BED in patients prescribed with D95 or ≥ 130 Gy BED in patients prescribed with an isocenter dose) as well in patients with rectal cancer, age ≥ 70 years old, and receiving adjuvant chemotherapy after SBRT in multivariate analysis.

A retrospective study including 577 eligible patients utilizing a patient registry analyzed OS in 447 patients and LC in 304 patients. The median OS was 26 months, with actuarial survival at 1, 3, and 5 years of 74.1%, 33.3%, and 21.8%, respectively. The median LC was 53 months and had rates of 80.4%, 58.9%, and 46.3%, respectively at the same intervals. Contrary to what was described by Sharma, colorectal primary had worse OS than the head and neck or breast primaries, but no differences were seen in LC [70]. Gomez et al. compared local consolidative therapy (LCT) using either radiation therapy or surgery versus observation in patients with NSCLC who did not progress after front-line systemic therapy. This study was closed early, with only 49 patients assigned after a significantly improved progression-free survival (PFS) was seen in the LCT arm. The PFS benefit was a median of 14.2 months in the intervention arm against 4.4 months in the observation arm (*p* = 0.022). OS had a median benefit of 24.2 months in the intervention arm compared with the observation arm (41.2 vs. 17 months) [29]. The SABR-COMET trial randomized 99 patients (with a life expectancy of at least 6 months) to receive palliative standard of care or standard of care and SABR. The SABR group had a preponderance of prostate cancer patients (21%), while the control group had a preponderance of colorectal cancer patients (27%). The median OS in the no-SABR group was 28 versus 41 months in the SABR group. However, there was a 20% increase in grade 2 or worse adverse events, and three patients out of 66 in the SABR treatment group had treatment-related deaths [9]. Although these two major clinical trials were not designed to evaluate the outcome of SBRT for lung metastases, many of the patients included in these two studies were treated with SBRT for lung metastases.

## 10. Acute and Late Toxicity

SBRT for pulmonary metastases, is usually devoid of significant toxicities, with less than 5% of patients featuring acute grade 2 or higher toxicity: In regards to late toxicity, the most-reported symptom in that study was grade 2 cough (7.5%) and grade 2 fatigue (6%) [106]. In a study of 207 patients, Kessel reported a higher incidence of grade 2 toxicity and a 9.7% rate of symptomatic pneumonitis [107].

Toxicities higher than grade 2 are low, with only 2.9% presenting symptoms within the first 6 months and 2.5% after 1 year. In Kessel’s study, patients who reported late severe dyspnea after the SBRT treatment had been diagnosed with chronic obstructive pulmonary disease before the treatment [107].

## 11. Prognostic Factors

In a series of 206 patients treated with SBRT and a median follow-up of 26 months, 54% of the patients died, the median OS was 33 months, and the median PFS was 13 months. Although the median PFS survival was low at 2, 3, and 5 years (36%, 25%, and 16%, respectively), the local control was high (85%, 83%, and 81%, respectively) [106].

In another series of 219 patients with a median follow-up of 16.5 months, the median OS was 27.6 months, and rates of distant progression (DP)-free survival at 2, 3, and 5 years (46%, 40%, and 34%, respectively) were also lower than LC at 2, 3, and 5 years (84%, 78%, and 75%, respectively) [107].

A retrospective study that included 577 eligible patients utilizing a patient registry analyzed OS in 447 patients and LC in 304 patients. The median OS was 26 months, with actuarial survival rates at 1, 3, and 5 years of 74.1%, 33.3%, and 21.8%, respectively. The median LC was 53 months with rates of 80.4%, 58.9%, and 46.3%, respectively at the same intervals. Contrary to what was described by Sharma [106], colorectal primary had worse OS than head and neck or breast primaries, but no differences were seen in LC [70]. Gomez et al. compared local consolidative therapy (LCT) using either radiation therapy or surgery versus observation in patients with oligometastatic NSCLC who did not progress after front-line systemic therapy. This study was closed early, with only 49 patients assigned after significantly improved progression-free survival (PFS) was seen in the LCT arm. The PFS benefit was a median of 14.2 months in the intervention arm against 4.4 months in the observation arm (*p* = 0.022). OS had a median benefit of 24.2 months in the intervention arm compared with the observation arm (41.2 vs. 17 months) [29]. The SABR-COMET trial randomized 99 patients with oligometastatic cancer at various locations (with a life expectancy of at least 6 months) to receive palliative standard of care or standard of care and SABR. The SABR group had a preponderance of prostate cancer patients (21%), while the control group had a preponderance of colorectal cancer patients (27%). The median OS in the no-SABR group was 28 versus 41 months in the SABR group. However, there was a 20% increase in grade 2 or worse adverse events, and three patients out of 66 in the SABR treatment group suffered treatment-related death [9].

## 12. Conclusions

- Lung SBRT is an external beam radiation therapy method that accurately delivers a high dose of radiotherapy within a limited number of fractions, often using biologically effective doses ≥ 100 Gy^10^.
- Its use in treating lung oligometastases is becoming increasingly prevalent with evidence supporting both a clinical benefit and limited toxicity.
- There is variation in the dose-fractionation schedules used, and an optimal regimen for central or ultracentral tumours has yet to be defined.
- The main technical requirements for SBRT include modern linear accelerators with image-guided radiation therapy, advanced immobilization devices, motion management strategies, and quality controls.

## Figures and Tables

**Table 1:** Patient eligibility criteria for SBRT.

Age	Any
ECOG	0–2
Medically operable patients	Patients who refuse surgical intervention
Number of lesions	Range 1–5
Tumor diameter	<50 mm
Location	Peripheral Central
Medically inoperable patients	Poor lung function: FEV1 < 40% predicted, postoperative FEV1 < 30% predicted, decrease diffusing capacity < 40% predicted, baseline hypoxemia (≤70 mm Hg) and/or hypercapnia (>50 mmHg), and oxygen consumption during exercise < 50% predicted. Important comorbidities: Severe pulmonary hypertension; diabetes mellitus with end-organ damage; severe cerebral, cardiovascular or peripheral vascular disease; or severe chronic heart disease

ECOG: Eastern. Operative Oncology Group Scale of performance status. FEV1: Forced Expiratory Volume in the first second.

**Table 2:** Brief Summary of published studies of lung SBRT-treated central and central/peripheral lung metastases and differences in the prescribed dose.

Author/Year	Location	Technique Description	Prescribed Dose	Local Control	Overall Survival	Grade > 3 Toxicity
Milano et al. 2009 [79]	Central	Relaxed end-expiratory breath holding	Dmean 50 Gy (30–63 Gy) most in 4–5 Gy per fx	73% at 2 yr	47% at 2 yr	5/53 pts w/grade 5
Unger et al. 2010 [83]	Central	CyberKnife system with synchrony fiducial tracking technology	30–40 Gy in 5 fx	63% at 1 yr	54% at 1 yr	3/20 pts w/severe pneumonitis
Rowe et al. 2012 [84]	Central 100%	4D-CT with ITV and CBCT guidance system	75% BED 100 Gy 57% 12.5 Gy × 4 fx 25% BED <100 Gy	75% at 2 yr	_______	5/47 patients
Nuyttens et al. 2012 [85]	Central	CyberKnife respiratory tumor tracking system	45–60 Gy/5–6 Fx	64% at 2 yr	75% at 2 yr	No grade 4–5 toxicity, 17.12% grade 3
Nuyttens et al. 2014 [86]	Peripheral Size >3 cm	Real-time tumor tracking + radiopaque markers	60 Gy/3 fx	90% at 2 yr	58% at 3 yr	No grade 4–5 toxicity
	Peripheral Size <3 cm		30 Gy/1 fx	74% at 2 yr		
	Central		60 Gy/5 fx	100% at 2 yr	53% at 3 yr	
	Central in contact with the esophagus or mediastinum.		56 Gy/7 fx	100% at 2 yr		
Chaudhuri et al. 2015 [80]	Central 50%	IMRT/4D-CT/PET respiratory gating	(78%) 50 Gy/4 fx; (22%) 50.4 Gy/5 fx. Proportionally, more centrally located with 5 fx.	_______	73.8% at 2 yr No differences regarding tumor location	3% at 3 yr
	Peripheral 50%			_______		11.6% at 3 yr
Davis et al. 2015 [76]	Central	CyberKnife with synchrony respiratory motion tracking system	Dmean 37.5 Gy (16–60 Gy) in 1–5 fx (media 3 fx), Dmean BED 93.6 Gy	69.8% at 2 yr	49.5% at 2 yr	No grade 3–5 toxicity
Haseltine et al. 2015 [87]	Central	4D-CT with ITV and CBCT guidance system	36–60 Gy in 2–5 fx, 56% received 45 Gy in 5 fx	77.4% at 2 yr	63.9% at 2 yr	12%, four patients with grade 5
Lischalk et al. 2016 [78]	Central	Synchrony respiratory motion tracking system with fiducial markers	35–40 Gy/5 fx BED 59.5–72 Gy	57.4% at 2 yr No differences regarding the prescribed dose	40% at 2 yr No differences regarding the prescribed dose	15% (one patient with grade 4)
Lindberg et al. 2017 [88]	Central ≤1 cm from the proximal bronchial tree	_______	56 Gy/8 fx	_______	_______	28% grade 3–5

ITV: Internal target volume; Dmean: Mean dose; BED: Biologically equivalent dose; Gy: Gray; fx: Fractions; and yr: Years.

**Table 3:** SBRT doses and fractionations for lung lesions according to the ESTRO ACROP consensus on the implementation and practice of SBRT for peripheral lesions in early-stage non-small-cell lung cancer.

Tumor Location	Dose to PTV	BED10 of the Prescribed Dose to the PTV
Peripheral	3 × 15 Gy (45 Gy)	113 Gy BED10
Central	4 × 12 Gy (48 Gy)	106 Gy BED10

**Table 4:** CARO clinical practice guidelines for lung SBRT.

Prescribed Dose for PTV	BED10 of the Prescribed Dose to the PTV
8 × 7.5 Gy (60 Gy)	105 Gy BED10
5 × 10 Gy (50 Gy)	100 Gy BED10
4 × 12 Gy (48 Gy)	106 Gy BED10
3 × 18–20 Gy (54–60 Gy)	151–180 Gy BED10
1 × 34 Gy (34 Gy)	150 Gy BED10
